# Mechanisms of the marine yeast *Debaryomyces hansenii* for protection against reactive oxygen species produced during benzo(a)pyrene biotransformation

**DOI:** 10.1128/aem.02314-25

**Published:** 2026-01-07

**Authors:** Francisco Padilla-Garfias, Minerva Georgina Araiza-Villanueva, Martha Calahorra, Norma Silvia Sánchez, Antonio Peña

**Affiliations:** 1 Departamento de Genética Molecular, Instituto de Fisiología Celular, Universidad Nacional Autónoma de México, Ciudad Universitaria 7180, Mexico City, Mexico; Universidad de los Andes, Bogotá, Colombia

**Keywords:** yeast, extremophilic yeasts, *Debaryomyces hansenii*, polycyclic aromatic hydrocarbons, benzo(a)pyrene, biotransformation, bioremediation, mycoremediation, xenobiotic metabolism, detoxification pathways, glutathione metabolism, oxidative stress

## Abstract

Polycyclic aromatic hydrocarbons (PAHs), such as benzo(a)pyrene (BaP), are persistent environmental pollutants recognized for their high toxicity and resistance to microbial degradation. The marine yeast *Debaryomyces hansenii* has emerged as a compelling candidate due to its ability to thrive under a variety of stressful conditions. This study presents a molecular and biochemical analysis of the responses of *D. hansenii* to BaP exposure, providing insights into the detoxification and antioxidant mechanisms. In this study, we explored BaP removal from the medium and characterized the associated molecular and biochemical responses induced by this compound. Our findings demonstrate that *D. hansenii* can grow in BaP concentrations up to 100 ppm without compromising cell viability and is capable of biotransforming nearly 70% of the compound within 6 days. This process involved cytochrome P450 (CYP), epoxide hydrolase (EH), and glutathione S-transferase (GST), enzymes typically associated with xenobiotic metabolism. BaP exposure resulted in pronounced oxidative stress, evidenced by elevated ROS levels, lipid peroxidation, and protein carbonylation. However, *D. hansenii* activated a robust antioxidant defense, including superoxide dismutase (SOD), catalase (CAT), glutathione peroxidase (GPx), and regulation of the glutathione redox system. Altogether, these results show that *D. hansenii* maintains redox homeostasis during BaP biotransformation through a coordinated enzymatic network that links xenobiotic metabolism with antioxidant protection, thereby expanding mechanistic understanding and providing a basis for future studies under environmentally relevant conditions.

**IMPORTANCE:**

Understanding how marine yeasts respond to polycyclic aromatic hydrocarbons (PAHs) is important for advancing our knowledge of stress adaptation and xenobiotic biotransformation in extremophilic microorganisms. *Debaryomyces hansenii* is a suitable model organism because it naturally tolerates high salinity, oxidative conditions, and nutrient limitation. By characterizing its responses to benzo(a)pyrene (BaP), we can gain insight into how eukaryotic microbes activate biotransformation pathways and antioxidant defenses under chemical stress. This insight contributes to a broader comprehension of cellular strategies for coping with toxic contaminants and provides the basis for future applied research.

## INTRODUCTION

Benzo(a)pyrene (BaP) is a five-ring polycyclic aromatic hydrocarbon (PAH) that originates from both natural events (such as volcanic activity) and human-driven processes (including fossil fuel combustion and tobacco use). It occurs widely in coal residues, petroleum sludge, asphalt, and cigarette smoke (1). Its high chemical stability underlies its strong environmental persistence, making it resistant to natural degradation and predisposing it to accumulation in soils and aquatic sediments (2). As a result, BaP is considered one of the most persistent and ubiquitous toxicants in both terrestrial and aquatic ecosystems (3).

Human exposure to BaP has increased in recent decades through combustion emissions and the consumption of contaminated foods (4). BaP is absorbed by inhalation, ingestion, or dermal contact and is metabolized by cytochrome P450 (CYP) enzymes into reactive intermediates. One of the intermediaries is notably BaP-7,8-diol-9,10-epoxide (BPDE) that exhibits carcinogenic, mutagenic, and teratogenic activities (1, 4). In addition to these classical toxic effects, BaP has been linked to neurotoxicity, epigenetic alterations, and reproductive damage, underscoring its broad impact on humans and environment (1, 4, 5).

Due to BaP’s persistence and toxicological relevance, there is a growing interest in sustainable remediation strategies. Among these, mycoremediation (the use of fungi to degrade or transform organic pollutants) has gained attention for its ecological compatibility and enzymatic versatility (6, 7). Although conventional approaches often rely on biochemical assays to measure fungal degradation capacity, the underlying metabolic and regulatory mechanisms remain poorly understood (7–9). Research in this field has predominantly focused on filamentous fungi from the Ascomycota and Basidiomycota phyla (10). In contrast, yeasts, which offer rapid growth, ease of genetic manipulation, and notable stress tolerance, have received limited attention despite their considerable biotechnological potential (11).

In Ascomycota fungi, including yeasts, BaP uptake occurs mainly through passive diffusion across the lipid bilayer due to its hydrophobic nature. This energy-independent process enables intracellular accumulation or transient storage of PAHs in lipid vesicles (12). Once inside, BaP undergoes a three-phase biotransformation: (i) aromatic ring oxidation by cytochrome P450 monooxygenases (CYP), their NADPH-dependent reductase (CPR), and epoxide hydrolases (EH); (ii) conjugation of reactive intermediates with functional groups such as glutathione, sulfate, or glucose, predominantly catalyzed by glutathione S-transferases (GST); and (iii) removal of the conjugates through vacuolar sequestration or active export (9, 12, 13). This multienzyme system, known as the xenome, represents the core machinery for fungal xenobiotic metabolism (9, 11, 14, 15). A comprehensive understanding of the molecular regulation and functional dynamics of this system is essential for advancing knowledge of fungal adaptation to toxic compounds and may ultimately lead to future applied studies.

A major challenge during BaP biotransformation is the oxidative stress that arises in the early oxidation steps. Reactive oxygen species (ROS) formed as by-products can damage lipids, proteins, and nucleic acids (16–18). To counteract this, fungi activate antioxidant defenses that involve superoxide dismutase (SOD), catalase (CAT), and glutathione peroxidase (GPx). Central to these defenses is reduced glutathione (GSH), a versatile tripeptide that neutralizes free radicals and maintains intracellular redox balance (19–21).

The marine yeast *Debaryomyces hansenii* has gained increasing attention for its ability to thrive under environmental conditions that inhibit most fungi, including high salinity, low temperatures, and elevated concentrations of heavy metals or oxidants such as H₂O₂ (22–24). Its persistence in these habitats is supported by a flexible metabolism capable of using diverse carbon sources, including hydrocarbons (22, 24–26), as well as by an efficient antioxidant system that maintains redox balance and preserves cellular components under stress (22). These physiological traits are consistent with its broad tolerance to salinity, temperature fluctuations, heavy metals, and oxidative agents, features that underpin its capacity to withstand chemically demanding environments (27–30). Building on this extremotolerant profile, *D. hansenii* has also been shown to metabolize PAHs; notably, BaP is transformed through a CYP enzyme encoded by *DhDIT2* (31).

Recently, transcriptomic studies suggested that *D. hansenii* activates a complex network of metabolic and stress-response pathways when exposed to BaP (26). These include oxidation by CYP and EH, antioxidant protection through glutathione-dependent and ROS-scavenging enzymes, conjugation and export of metabolites, catabolism, and membrane remodeling. Although these transcriptomic predictions outlined an integrated metabolic framework, the biochemical evidence supporting them has remained limited.

This study, therefore, focuses on the functional mechanisms that allow *D. hansenii* to metabolize BaP and withstand the oxidative stress generated during its transformation. Through a combination of enzyme activity assays, redox markers, and gene expression analysis, we characterized how biotransformation and antioxidant defenses are coordinated over time. These results offer a comprehensive framework for understanding the adaptive strategies of *D. hansenii* and highlight its potential relevance for future environmental applications.

## MATERIALS AND METHODS

### Strain and cultures

*Debaryomyces hansenii* Y7426 strain (United States Department of Agriculture, Peoria, Illinois, USA) was routinely maintained on solid YNBG medium and refreshed once a month (yeast nitrogen base plus glucose). The medium contained 0.67% (wt/vol) yeast nitrogen base without amino acids (Bio Basic Inc., Cat. S507), 2% (wt/vol) glucose, 2% (wt/vol) agar, and the following amino acid supplements: histidine (20 mg/L), methionine (20 mg/L), tryptophan (20 mg/L), leucine (30 mg/L), and lysine (100 mg/L). The defined YNB formulation consisted of 5.0 g/L (NH_4_)_2_SO_4_, 1.0 g/L KH_2_PO_4_, 0.5 g/L MgSO_4_, 0.1 g/L NaCl, 0.1 g/L CaCl_2_, trace elements (H_3_BO_3_ 0.5 mg/L, CuSO_4_ 0.04 mg/L, KI 0.1 mg/L, FeCl_3_ 0.2 mg/L, MnSO_4_ 0.4 mg/L, Na_2_MoO_4_ 0.2 mg/L, ZnSO_4_ 0.4 mg/L), and vitamins (biotin 0.002 mg/L, folic acid 0.002 mg/L, inositol 2 mg/L, niacin 0.4 mg/L, p-aminobenzoic acid 0.2 mg/L, calcium pantothenate 0.4 mg/L, pyridoxine-HCl 0.4 mg/L, riboflavin 0.2 mg/L, and thiamine-HCl 0.4 mg/L).

Cultures were propagated in 500 mL Erlenmeyer flasks containing 250 mL of liquid YNBG (same composition without agar). Incubations were carried out at 28°C with orbital shaking at 250 rpm for 24 h.

For BaP exposure, a 10,000 ppm (10 mg/mL) stock solution was prepared by dissolving 10 mg of benzo(a)pyrene (Sigma-Aldrich, USA; Cat. B1760) in 1.0 mL of analytical-grade acetone. The stock was added to the culture medium to a final concentration of 100 ppm (100 µg/mL; ~396 µM). The final acetone concentration was kept below 1% (vol/vol), a condition previously verified to have no effect on the growth or metabolism of *D. hansenii* (31). Experimental cultures were initiated at an optical density at 600 nm (OD_600_) of 0.1.

### Growth and survival assays

Growth and survival assays were conducted under aseptic conditions using three independent biological replicates, each analyzed in technical duplicates. The growth conditions (28°C, 100 ppm BaP, 6 days) were previously established for this yeast in a medium with identical composition (31).

For growth on solid media, cells were pre-cultured for 24 h in YNBG, washed twice with sterile water, and adjusted to an OD_600_= 1.0. Ten-fold serial dilutions were prepared in sterile water in 96-well plates, and 5 µL of each dilution was spotted onto YNBG (control) and YNB + 100 ppm BaP (glucose-free) agar plates using a multi-pin replicator. Plates were incubated at 28°C and monitored daily for 6 days.

For growth curves analysis, 24-h pre-cultures were adjusted to OD_600_ of 0.03, using a Beckman DU 650 spectrophotometer and inoculated into the respective media (YNBG or YNB + 100 ppm BaP). Cultures were incubated at 28°C, and OD_600_ was recorded hourly for 6 days using a Bioscreen C automated plate reader (with an initial OD_600_ baseline of approximately 0.2).

For biomass quantification, pre-cultures were used to inoculate 50 mL of YNBG or YNB + 100 ppm BaP at OD_600_ = 0.1. At 24-h intervals, 1-mL aliquots were filtered through pre-weighed 0.22 µm membranes (Merck Millipore, Sampling Manifold 1225), washed twice with sterile water, and dried at 95°C until constant weight. Final dry weights were recorded on an analytical balance.

### BaP removal assay

BaP removal was quantified by spectrofluorometry, following Padilla-Garfias et al. (31) validated by Gas Chromatography–Mass Spectrometry (GC-MS) in earlier studies (26, 31). All assays were performed by triplicate with three independent biological replicates. Cultures of *D. hansenii* pre-grown for 24 h in YNBG were washed twice with sterile water and used to inoculate 250 mL of YNB + 100 ppm BaP, adjusted to an initial OD_600_ = 0.1. Samples (3.0 mL) were collected at 0, 1, 2, 3, and 6 days and stored at −20°C for BaP extraction.

Residual BaP was extracted by mixing each sample three times with 3.0 mL chloroform, followed by vortexing and centrifugation at 3,000 rpm (1,750 × *g*) for 5 min (IEC clinical centrifuge) to separate phases. The organic phase was recovered, evaporated at 62°C, and the residue resuspended in 3.0 mL acetone. Fluorescence was measured using an AMINCO SLM spectrofluorometer (excitation wavelength at 356 nm, emission wavelength at 405 nm), and concentrations were calculated from a standard curve prepared under identical solvent conditions.

To account for non-biological loss of BaP, two negative controls were included: (i) a cell-free medium control and (ii) a heat-inactivated biomass control (95°C, 15 min) to distinguish between biological degradation from physical adsorption.

### RNA extraction

*D. hansenii* cells were initially grown in 250 mL of YNBG medium at 28°C with shaking at 250 rpm for 24 h. After this pre-culture, cells were harvested, washed twice with sterile water, and 50 mL flasks containing YNBG or YNB + 100 ppm BaP at an OD_600_ = 0.1 were inoculated and incubated under the same conditions for 0, 1, 2, 3, and 6 days.

For RNA extraction, 15 mL of culture was collected from each sampling day. Cells were pelleted by centrifugation (5 min at 3,000 rpm, 1,750 × *g*) and resuspended in 1.0 mL of AE buffer (50 mM sodium acetate, 10 mM EDTA, pH 5.2). Total RNA was extracted using a modified phenol–SDS method (32). Briefly, 40 μL of 10% SDS, 450 μL of acid phenol (pH 4.3), and 400 μL of 0.45 mm diameter glass beads were added to the suspension, followed by vigorous vortexing and incubation at 65°C for 20 min with intermittent mixing. Samples were then snap-frozen in liquid nitrogen for 3 min and centrifuged at 10,000 rpm (6,700 × *g*) for 10 min. The aqueous phase was recovered and further purified with two sequential extractions using phenol/chloroform/isoamyl alcohol (25:24:1) and a final extraction with chloroform/isoamyl alcohol (24:1). RNA was precipitated with 0.3 M sodium acetate and 2.5 volumes of absolute ethanol, washed with 70% ethanol, air-dried, and dissolved in nuclease-free water. RNA integrity was confirmed by 1% denaturing agarose gel electrophoresis, showing intact 28S and 18S rRNA bands.

### Gene expression studies: cDNA synthesis and RT-qPCR

Total RNA was treated with DNase I (RQ1 RNase-Free DNase kit, Promega) to remove genomic DNA contamination and reverse transcribed into cDNA using the ImProm-II Reverse Transcription System (Promega). Gene expression was analyzed by RT-qPCR using four independent biological replicates and two technical replicates per sample. 

RT-qPCR was performed using specific primers (see Table S1) targeting 24 open reading frames (ORFs) identified as upregulated in RNA-Seq data sets deposited in the NCBI Gene Expression Omnibus (accession no. GSE299919) (26). The corresponding gene orthologs were identified in the MycoCosm database (https://mycocosm.jgi.doe.gov/mycocosm/home) (33) (see Table S2).

Sequences were retrieved from NCBI, and primers were designed with Primer-BLAST and then evaluated for dimer formation and specificity using DINAMelt (http://www.unafold.org/hybrid2.php) (34). Primer synthesis was performed at the Molecular Biology Unit of the Institute of Cellular Physiology, UNAM. qPCRs were run on a Rotor-Gene Q thermal cycler (Qiagen) using SYBR Green chemistry (qPCR SyberMaster highROX, Jena Bioscience). Relative gene expression levels were calculated by the 2⁻^ΔΔCT^ method (35), with YNBG-grown cells as the calibrator. Expression values were normalized to *DhACT1* (ORF DEHA2D05412g), as described by Sánchez et al. (27).

### Preparation of protein extracts

Cells were pre-cultured for 24 h in YNBG, harvested, washed twice with sterile water to minimize carry-over of glucose, and then used to inoculate 250 mL of YNBG (control) or YNB supplemented with 100 ppm BaP, adjusted to an initial OD_600_= 0.1. Cultures were incubated at 28°C with shaking (250 rpm) and sampled on days 0, 1, 2, 3, and 6.

Cells were collected by centrifugation for 5 min at 3,000 rpm (1,400 × *g*) in a Beckman Coulter Avanti J-26 XPI centrifuge. Pellets were resuspended at 1 g/mL (wet weight/volume) in 10 mM 3-(N-morpholino) propanesulfonic acid (MOPS) buffer (pH 7.0) supplemented with protease inhibitors (cOmplete, Roche). Glass beads (0.45 mm diameter) were added to half the sample volume, and cells were disrupted using a Bead Beater chamber placed on ice, performing six 30-s homogenization cycles with 2-min intervals between each one. All steps were carried out at 4°C. Cell debris was removed by centrifugation at 3,000 rpm (1,750 × *g*) for 5 min in an IEC clinical centrifuge, and the clarified supernatants were used for subsequent enzymatic assays.

### Enzymatic activities

Prior to the enzymatic assays, the clarified supernatants obtained from cell extracts (section 2.6) were centrifuged at 10,000 rpm (6,700 × *g*) for 10 min in an Eppendorf 5415C centrifuge. Protein concentration was determined by the Lowry method modified by Markwell (36) using a Beckman DU 650 spectrophotometer. All enzymatic activities were subsequently measured with an Aminco DW-2a UV/VIS spectrophotometer (OLIS, On-Line Instrument Systems, Inc., converted to DW2).

The activity of cytochrome P450 (CYP) was indirectly estimated by measuring the NADPH-cytochrome *c* reductase (CPR), which functions as the electron donor protein for CYP-mediated reactions. CPR activity was quantified using the Cytochrome *c* Reductase (NADPH) Assay Kit (CY0100, Sigma, Saint Louis, MO, USA), following the manufacturer’s instructions. Enzyme activity values were normalized to protein content (mg).

Epoxide hydrolase (EH) activity was assayed according to Mateo et al. (37), with minor modifications (37). Reactions were carried out in 3 mL mixtures containing 100 mM sodium phosphate buffer (pH 7.0), 2 mM styrene oxide (dissolved in dimethylformamide), 2 mM sodium metaperiodate, and 40 µg of protein extract. Enzymatic activity was monitored by the increase in absorbance at 290 nm for 2 min at 30°C. Specific activity was calculated using the molar extinction coefficient of benzaldehyde (*ε* = 1.34 mM⁻¹ cm⁻¹).Glutathione S-transferase (GST) activity was determined following the method of Habig and Jakoby (38), with minor modifications (38). Each 2 mL reaction mixture contained 100 mM sodium phosphate buffer (pH 6.5), 5 mM reduced glutathione (GSH), 1 mM 1-chloro-2,4-dinitrobenzene (CDNB; dissolved in ethanol), and 40 µg of protein extract. The formation of the GSH–CDNB conjugate was monitored by the increase in absorbance at 340 nm for 3 min at 30°C. Specific activity was calculated using the molar extinction coefficient of the adduct (*ε* = 9.6 mM⁻¹ cm⁻¹).

Glutathione reductase (GR) activity was estimated according to Carlberg and Mannervik (39), with minor modifications (39). Each 2 mL reaction mixture contained 100 mM sodium phosphate buffer (pH 7.0), 1 mM oxidized glutathione (GSSG), 0.15 mM NADPH, and 40 µg of protein extract. The decrease in absorbance at 340 nm, corresponding to NADPH oxidation, was monitored for 2 min at 30°C. Specific activity was calculated using the extinction coefficient of NADPH (*ε* = 6.2 mM⁻¹ cm⁻¹).

Glutathione peroxidase (GPx) activity was assayed following González et al. (40), with minor modifications (40). Each 2 mL reaction mixture contained 100 mM sodium phosphate buffer (pH 7.0), 1 mM reduced glutathione (GSH), 0.15 mM NADPH, 1 unit of glutathione reductase (GR), 1 mM hydrogen peroxide (H₂O₂), and 40 µg of protein extract. The decrease in absorbance at 340 nm, reflecting NADPH consumption, was monitored for 1 min at 30°C. Specific activity was calculated using the extinction coefficient of NADPH (*ε* = 6.2 mM⁻¹ cm⁻¹).

Superoxide dismutase (SOD) activity was measured according to González et al. (40), with minor modifications (40). Each 2 mL reaction mixture contained 50 mM sodium phosphate buffer (pH 7.0), 0.1 mM EDTA, 13 mM methionine, 75 µM nitroblue tetrazolium (NBT), 2 µM riboflavin, and 40 µg of protein extract. Control reactions contained equivalent volumes of buffer in place of protein. The mixtures were exposed to fluorescent light (UVP transilluminator) for 10 min to initiate the reaction, followed by a 10 min incubation in the dark. Absorbance was measured at 560 nm. SOD activity was expressed as the enzyme amount required to inhibit 50% of NBT reduction, using the formula:


$$SOD\;activity\left(\frac{U}{mg\;protein}\right)=\left(\frac{\Delta {Abs}_{control}-\Delta {Abs}_{sample}}{\Delta {Abs}_{control}}\right)\times 100$$

Catalase (CAT) activity was determined according to Aebi (41), with minor modifications (41). Each reaction mixture contained 100 mM sodium phosphate buffer (pH 7.0) and 1 mM hydrogen peroxide (H₂O₂). The decomposition of H₂O₂ was monitored by measuring the decrease in absorbance at 240 nm for 2 min at 30°C. Specific CAT activity was calculated from a calibration curve prepared with H₂O₂ concentrations ranging from 1 mM to 10 mM.

### Quantification of reduced glutathione and oxidized glutathione

For glutathione quantification, the supernatants from cell extracts prepared as described in “Preparation of protein extracts” and “Enzymatic activities,” above, were used; concentrations were normalized to the total protein content and expressed as µmol of GSH or GSSG per mg of protein.

Glutathione levels were determined following the method of Griffith (42). Extracts were deproteinized with 300 mM phosphoric acid, incubated at 4°C for 20 min, and centrifuged at 10,000 rpm (6,700 × *g*) for 10 min in an Eppendorf centrifuge 5415C. GSH levels were quantified based on its reaction with DTNB (5,5′-dithiobis-(2-nitrobenzoic acid)) at 412 nm. A standard calibration curve was prepared using serial dilutions of GSH ranging from 0 to 100 µM to calculate sample concentrations.

To measure GSSG, free GSH was first derivatized with 2-vinylpyridine. GSSG was then reduced to GSH using GR and NADPH, and the absorbance of the resulting DTNB-GSH complex was measured at 412 nm. A separate standard curve was generated using GSSG standards (0–10 µM), subjected to the same derivatization and reduction steps.

### Quantification of oxidative stress markers

Intracellular reactive oxygen species (ROS) accumulation was monitored over 6 days using two fluorescence-based probes with different subcellular specificities. Cytoplasmic ROS were estimated with 10 µM 2′,7′-dichlorodihydrofluorescein diacetate (H₂DCF-DA), while mitochondrial ROS were assessed with 5 µM dihydrorhodamine 123 (DHR123) (43). Cells grown in YNBG (control) or YNB + 100 ppm BaP (experimental) were harvested daily, washed twice with sterile water, and incubated with the probes for 30 min at 30°C in the dark. Fluorescence was recorded in a POLARstar Omega microplate reader (BMG Labtech, Germany) at excitation/emission wavelengths of 485/535 nm (H₂DCF-DA) and 500/536 nm (DHR123). Signal intensities were normalized to cell density (OD₆₀₀) measured in the same device (44). These assays provide relative estimates of ROS accumulation and do not discriminate between individual species.

Lipid peroxidation was evaluated daily by quantifying malondialdehyde (MDA), a stable end-product of polyunsaturated fatty acid oxidation. Cell extracts were prepared as described in “Preparation of protein extracts,” above, deproteinized with 20% (wt/vol) trichloroacetic acid (TCA), incubated on ice for 10 min, and centrifuged at 10,000 rpm (6,700 × *g*, 10 min, 4°C). The supernatant was mixed with thiobarbituric acid (TBA) and heated for 60 min at 95°C. After cooling, the absorbance of the MDA–TBA adduct was measured at 532 nm (Beckman DU 650 spectrophotometer). MDA concentrations were calculated using the molar extinction coefficient (*ε* = 156 mM⁻¹ cm⁻¹) and normalized to total protein content (45). 

Protein oxidation was assessed daily by quantifying carbonyl groups. Cell extracts were prepared as described in “Preparation of protein extracts” and “Enzymatic activities,” above, and supernatants were incubated with 5 mM 2,4-dinitrophenylhydrazine (DNPH) for 1 h at room temperature in the dark with occasional mixing. Protein hydrazones were precipitated with 20% TCA, washed with ethanol/acetone (1:1, vol/vol), and resolubilized. Absorbance was measured at 370 nm (Beckman DU 650 spectrophotometer). Carbonyl content was calculated using the molar extinction coefficient (*ε* = 22 mM⁻¹ cm⁻¹) and expressed relative to total protein (46).

### Statistical analysis

Statistical analyses were performed using GraphPad Prism 10 (GraphPad Software Inc., San Diego, CA, USA). Data were presented as mean ± standard deviation (SD) from three independent biological replicates. For some graphs, the area under the curve (AUC) was calculated and used for subsequent statistical analyses.

A standard one-way ANOVA was applied when comparing more than two groups under the assumptions of normal distribution and equal variances. Then, Tukey’s multiple comparisons test was performed with *P*-value multiplicity adjustment. Normality was assessed using the Shapiro–Wilk test, and homogeneity of variance was evaluated via Brown–Forsythe and Bartlett’s tests, all based on residual analysis using Prism’s built-in tools. For comparisons between two groups, an unpaired Student *t*-test was applied, assuming equal variances. Differences were considered statistically significant at *P* < 0.05. Exact *P*-values and significance levels were reported in the corresponding figure legends.

## RESULTS

To characterize how *D. hansenii* responds to BaP exposure, we combined growth assays, BaP removal measurements, targeted gene expression analyses, enzymatic activity assays, and oxidative stress markers. This multi-level approach allowed us to examine the temporal coordination between xenobiotic biotransformation and antioxidant defenses under controlled laboratory conditions.

**Fig 1:**
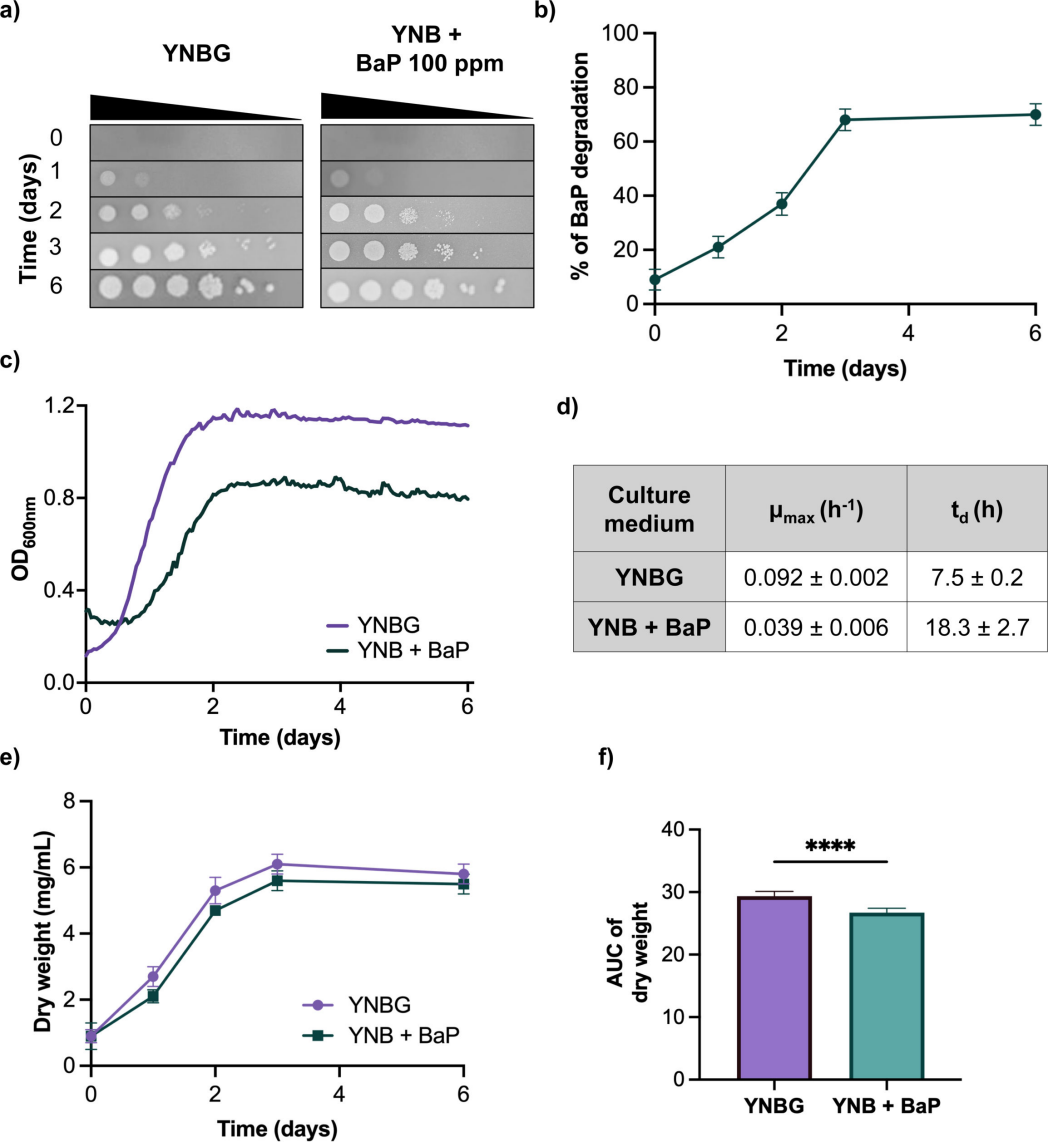
Growth and degradation capacity of *D. hansenii* in response to BaP exposure. (**a**) Spot assay on solid YNBG and YNB + 100 ppm BaP plates assessed by serial dilutions after 6 days of incubation at 28°C (representative image of *n* = 9). (**b**) BaP removal measured by fluorescence-based detection over 6 days (*n* = 9, three biological and three technical replicates). (**c**) Growth curves of *D. hansenii* in YNBG and YNB + 100 ppm BaP at 28°C. Optical density at 600 nm (OD_600_) was recorded every 30 min using a microplate reader (*n* = 30, 3 biological and 10 technical replicates). (**d**) Specific growth rate (*μ*_max_) and doubling time (*t*_d_) calculated from the exponential phase of growth curves shown in (**c**). (**e**) Biomass accumulation over six days, measured as dry weight (*n* = 18, three biological and six technical replicates). (**f**) Area under the curve (AUC) of biomass production was calculated for statistical comparison. Significance was assessed using two-tailed unpaired Student’s *t*-test (*P* < 0.05, ****P < 0.0001).

Assays showed that *D. hansenii* grows in the presence of 100 ppm of BaP in both solid and liquid media (Fig. 1a and c) and fluorescence-based quantification indicated ~70% signal decrease over 6 days (Fig. 1b) consistent with earlier observations (26, 31).

In liquid culture, doubling time increased from ~7.5 h (glucose) to ~18.3 h (BaP) (Fig. 1c and d). Biomass accumulation remained below the glucose condition across the time course (Fig. 1e), and area under the curve (AUC)-based comparisons confirmed significant differences between conditions (Fig. 1f).

After confirming growth and BaP removal, we used day-3 RNA-Seq data no. GSE299919 (26) to select 24 ORFs linked to detoxification, glutathione homeostasis, and antioxidant defenses (Table S2). We designed targeted primers (Table S1) and performed RT-qPCR at days 0, 1, 2, 3, and 6 under control and BaP conditions; heatmaps in Results summarize the complete datasets (Fig. S1).

**Fig 2:**
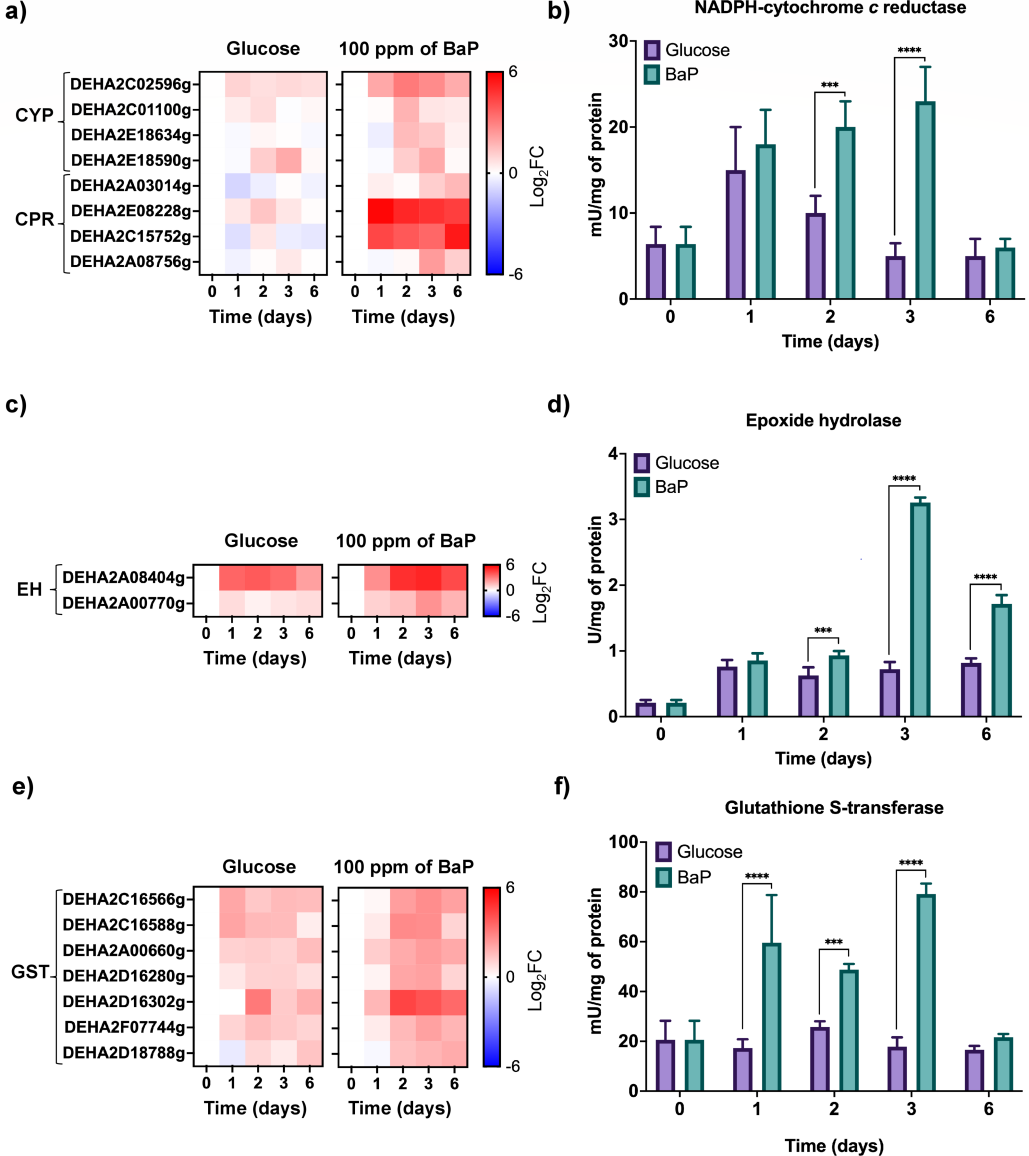
Differential gene expression and enzymatic activity of detoxification enzymes during BaP degradation in *D. hansenii*. (**a**) Heatmap of cytochrome P450 (CYP) and cytochrome P450 reductase (CPR) gene expression under control (YNBG) and YNB + BaP (100 ppm)-treated conditions. (**b**) NADPH-cytochrome *c* reductase activity measured over 6 days of growth. (**c**) Heatmap of epoxide hydrolase (EH) gene expression under the same conditions. (**d**) EH enzymatic activity over time. (**e**) Heatmap of glutathione S-transferase (GST) gene expression. (**f**) GST enzymatic activity profile. All RT-qPCR experiments were conducted with eight replicates (*n* = 8; four biological and two technical ones per condition), and expression values were normalized to *ACT1* (ORF DEHA2D05412g), control (YNBG) and YNB + 100 ppm BaP. Gene expression was quantified by RT-qPCR, normalized to *ACT1* (ORF DEHA2D05412g), using eight replicates (*n* = 8; four biological and two technical ones per condition). Heat maps were performed with data from Fig. S1. Enzymatic activities were assessed in three biological and three technical replicates (*n* = 9). Statistical analysis was performed using one-way ANOVA followed by Dunnett’s multiple comparisons test (*P* < 0.05). Asterisks indicate significance relative to control: (***) *P* = 0.0002; (****) *P* <0.0001.

Exposure to BaP induces the coordinated activation of detoxification enzymes in *D. hansenii*, as shown by the upregulation of CYP, CPR, EH, and GST ORFs and their corresponding activities (Fig. 2). CYP and CPR participate in BaP oxidation (phase I), EH converts the resulting epoxides into diols (phase II), and GST conjugates GSH to these intermediates (phase III) (11, 47, 48). All three enzymes exhibited maximal activity on day 3 under BaP treatment, coinciding with the highest compound transformation rate (Fig. 1b). Activity remained stable in glucose controls, indicating a BaP-specific response. A minor early increase of CPR and EH on day 1 likely reflects a non-specific cellular adjustment (5, 47–49).

**Fig 3:**
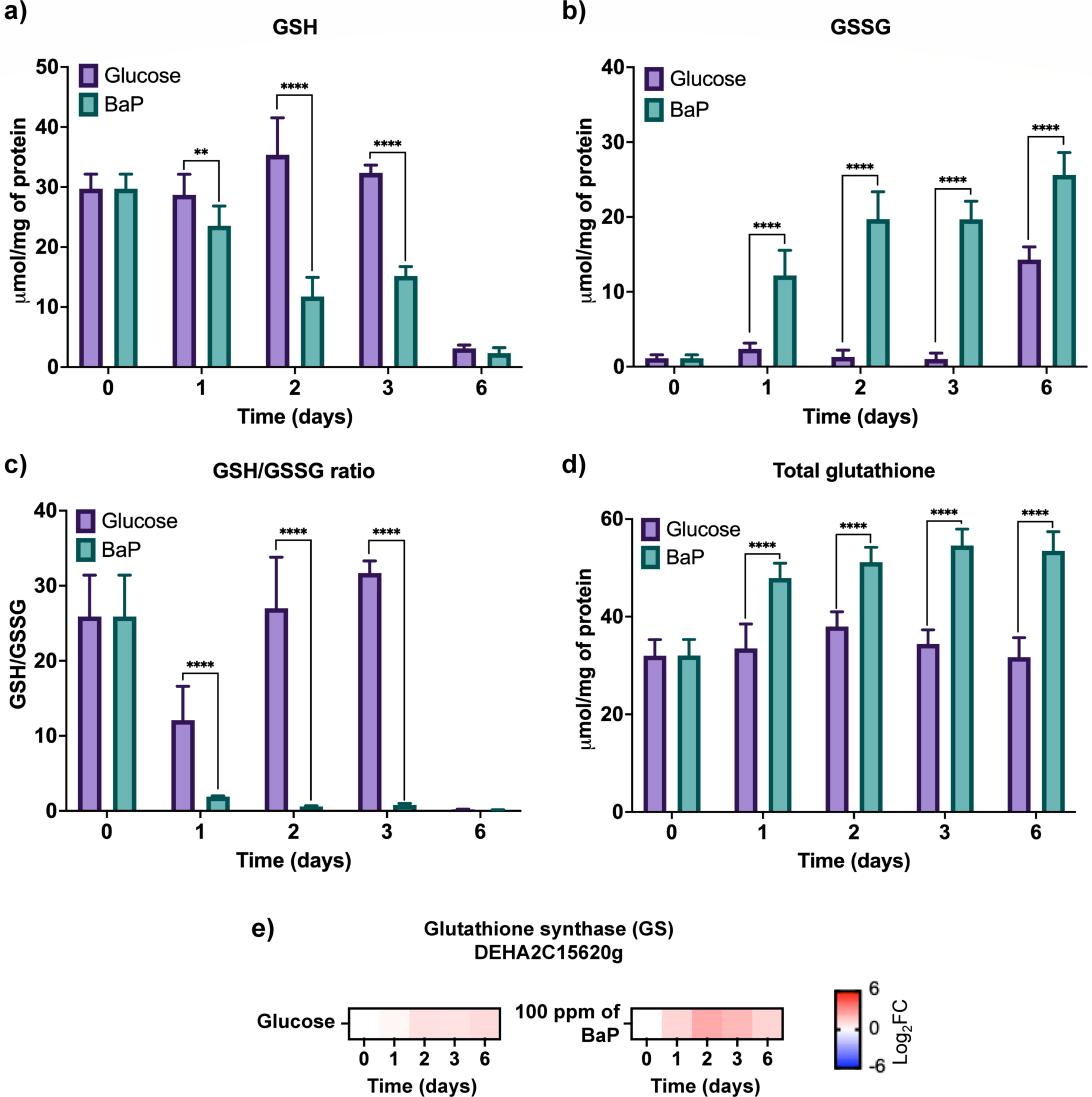
Changes in glutathione metabolism of *D. hansenii* in the presence of BaP. (**a**) Reduced glutathione (GSH) levels along 6 days of growth in control (YNBG) and BaP-treated (YNB + 100 ppm BaP) conditions. (**b**) Oxidized glutathione (GSSG) levels in the same conditions. (**c**) GSH/GSSG ratio as a redox stress indicator, calculated from panels (**a**) and (**b**). (**d**) Total glutathione content calculated as GSH + 2 × GSSG. (**e**) Relative expression of the glutathione synthetase (GS)-encoding ORF, measured by RT-qPCR. All biochemical measurements data were obtained from three biological and three technical replicates (*n* = 9). Gene expression was quantified by RT-qPCR and normalized to *ACT1* (ORF DEHA2D05412g), using eight replicates (*n* = 8; four biological and two technical ones per condition). The heat map was performed with data from Fig. S1. Statistical significance was determined by one-way ANOVA followed by Dunnett’s multiple comparisons test (*P* < 0.05). Asterisks indicate significance relative to control: (**) *P* = 0.0021; (****) *P* < 0.0001.

BaP exposure significantly increased the expression of GST-encoding ORFs and their enzymatic activity (Fig. 2e and f), suggesting that a large fraction of GSH was being consumed during conjugation. To evaluate the impact on redox balance, intracellular GSH and GSSG were quantified, total glutathione (GSH + 2×GSSG) was calculated, and the GSH/GSSG ratio was used as a redox indicator. We also measured the expression of glutathione synthetase (GS) to assess whether *de novo* synthesis of GSH was induced as a compensatory response (Fig. 3).

Exposure to BaP led to a marked decline in intracellular GSH levels, particularly by day 6 (Fig. 3a), consistent with its active consumption in conjugation and antioxidant defense. In parallel, GSSG levels increased significantly (Fig. 3b), and the GSH/GSSG ratio dropped (Fig. 3c), confirming an oxidative environment and the engagement of the glutathione pool to maintain redox balance. Despite this consumption, the total glutathione pool (GSH + 2×GSSG) rose steadily (Fig. 3d), accompanied by upregulation of glutathione synthetase (GS) expression (Fig. 3e). This coordinated pattern suggests that *D. hansenii* compensated for oxidative stress by stimulating de novo glutathione synthesis.

**Fig 4:**
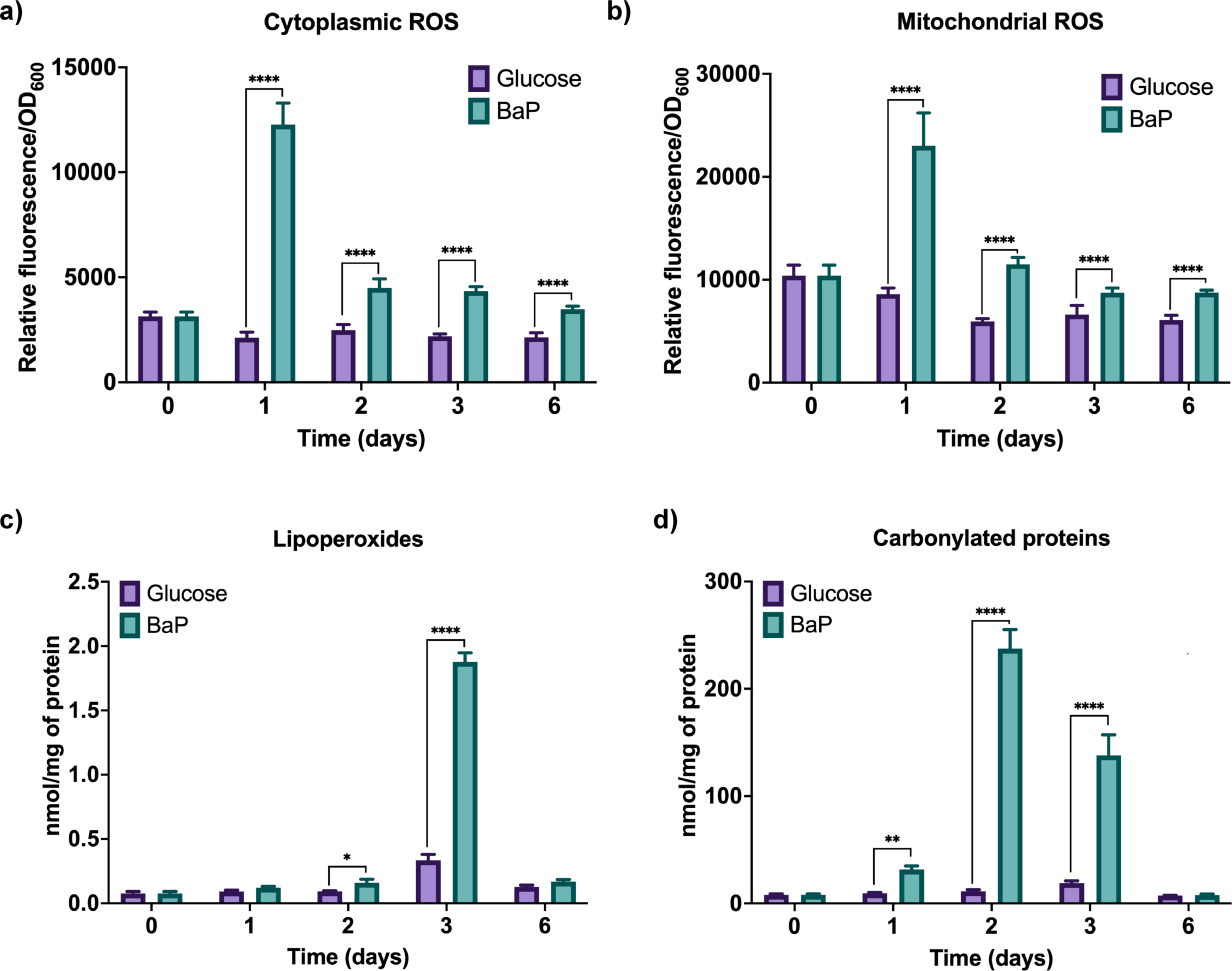
BaP exposure induces oxidative stress and molecular damage in *D. hansenii*. (**a**) Cytoplasmic reactive oxygen species (ROS) levels detected using the H₂DCF-DA (2′,7′-dichlorodihydrofluorescein diacetate) probe during 6 days of growth in YNBG (control) and YNB + 100 ppm BaP. (**b**) Mitochondrial ROS accumulation assessed using the DHR123 (Dihydrorhodamine 123) probe under the same conditions. (**c**) Lipid peroxidation levels measured as the malondialdehyde (MDA) content, indicating membrane damage. (**d**) Carbonylated proteins levels as a marker of oxidative protein damage. ROS levels were measured by fluorescence (H_2_DCF-DA: 485/535 and DHR123: 500/536 nm) and normalized to cell density (OD_600_). MDA and carbonylated proteins content were quantified spectrophotometrically (532 nm and 370 nm, respectively) and normalized to total protein. All experiments were performed using three biological and three technical replicates (*n* = 9). Statistical significance was determined using one-way ANOVA followed by Dunnett’s multiple comparisons test (*P* < 0.05). Asterisks indicate significance relative to control: (*) *P* = 0.0332; (**) *P* = 0.0021; (****) *P* < 0.0001.

Considering the alterations in glutathione homeostasis, the next step was to examine the accumulation of ROS. BaP exposure caused a rapid increase in both cytoplasmic and mitochondrial ROS (Fig. 4a and b), detectable from day 1. The dual localization indicates that oxidative stress originated from multiple cellular sources, likely linked to oxygen-consuming enzymes such as CYP monooxygenases. Fig. S2 shows that supplementation with GSH, MitoTEMPO, or ascorbate individually reduced BaP-induced ROS by ~50%, whereas combined treatments restored basal levels, confirming the mitochondrial–cytoplasmic origin of oxidative stress.

To assess the ROS accumulation damage, we quantified lipid peroxidation and protein carbonylation (Fig. 4c and d). Both indicators were significantly higher under BaP exposure, revealing oxidative injury to membranes and structural proteins.

**Fig 5:**
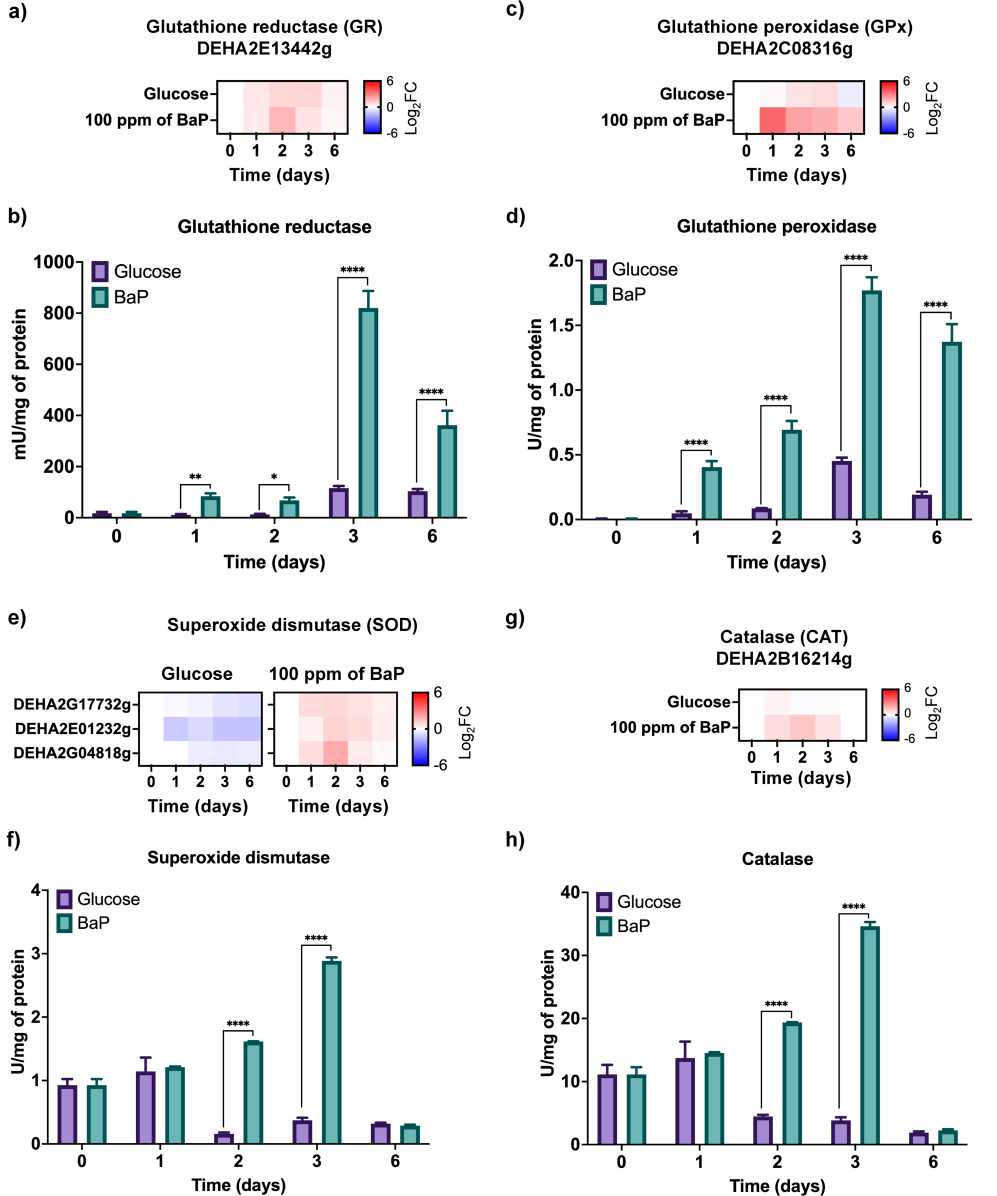
Activation of antioxidant defense enzymes in response to BaP-induced oxidative stress in *D. hansenii*. (**a**) Relative expression of glutathione reductase (GR) measured by RT-qPCR. (**b**) GR enzymatic activity across a 6-day time course. (**c**) Glutathione peroxidase (GPx) gene expression profile. (**d**) GPx enzymatic activity. (**e**) Expression of superoxide dismutase (SOD)-encoding ORFs. (**f**) SOD enzymatic activity. (**g**) Catalase (CAT) gene expression profile under control and BaP conditions. (**h**) Catalase enzymatic activity. RT-qPCR was performed with eight replicates (*n* = 8; four biological and two technical ones per condition), using *ACT1* (ORF DEHA2D05412g) for normalization. Heat maps were performed with data from Fig. S1. Enzymatic activity assays were conducted using three biological and three technical replicates (*n* = 9). Statistical significance was assessed by one-way ANOVA followed by Dunnett’s multiple comparisons test (*P* < 0.05). Asterisks indicate differences relative to control: (*) *P* = 0.0332; (**) *P* = 0.0021; (****) *P* < 0.0001.

The changes in glutathione levels, ROS accumulation, lipoperoxides, and carbonylated proteins led us to analyze antioxidant enzymes directly involved in redox recovery. A significant increase in GR transcript levels was observed on day 2 (Fig. 5a), accompanied by a strong rise of enzymatic activity (Fig. 5b). In parallel, GPx, which detoxifies organic peroxides, showed early transcriptional activation beginning on day 1 (Fig. 5c), along with an increased enzymatic activity (Fig. 5d). This response coincided with an elevated lipid peroxidation, underscoring the intense oxidative pressure experienced by the cells during BaP metabolism.

BaP exposure also activated the enzymatic defenses that directly eliminate reactive oxygen species. SOD, which catalyzes the conversion of superoxide radicals (O_2_•⁻) into H_2_O_2_, exhibited a strong transcriptional upregulation and increased enzymatic activity under BaP treatment (Fig. 5e and f). Similarly, CAT, responsible fodegrading H_2_O_2_ into H_2_O and O_2_, showed increased expression and activity (Fig. 5g and h). In summary, BaP exposure triggered a concerted response in *D. hansenii* involving the activation of multiple enzymatic systems, including CYP, CPR, EH, GST, GS, GR, GPx, SOD, and CAT. Together, these enzymes mediate BaP oxidation, conjugation, and ROS detoxification, sustaining redox balance throughout the exposure period. Although oxidative markers such as lipid peroxidation and protein carbonylation remained elevated, the coordinated activation of detoxification and antioxidant pathways demonstrates the yeast’s capacity to adjust its metabolism under BaP-driven oxidative stress.

## DISCUSSION

In contrast to previous studies that primarily addressed either gene identification or degradation rates, the present work provides an integrated view of *D. hansenii* responses to BaP, combining transcriptional, biochemical, and physiological evidence. Our findings reveal a tight coordination between the enzymatic systems responsible for BaP biotransformation and those mitigating the resulting oxidative stress, supporting the concept of a coupled biotransformation and antioxidant network (26, 31, 50–52). Unlike previous reports, this study provides functional and bichemical validation of transcriptomic predictions. Our results confirm the link between xenobiotic biotransformation and antioxidant regulation in *D. hansenii*. Altogether, this integrative evidence unveils a detoxification–redox interface that had not been previously described in marine yeasts.

*D. hansenii* can grow in both liquid and solid media in the presence of 100 ppm BaP, without loss of viability, and removed nearly 70% of the compound within 6 days, even under glucose-limited conditions. Although BaP is poorly soluble, its co-solubilization with acetone allowed uniform dispersion and bioavailability in the medium, a strategy previously applied in fungal biodegradation studies (26, 31, 53, 54). On the other hand, bacteria and yeasts like *D. hansenii* enhance hydrocarbon biodegradation by using biosurfactants and extracellular enzymes that increase the solubility of hydrocarbons such as PAHs and facilitate their adhesion to cells, thereby reducing interfacial energy (55). The decrease in BaP levels monitored by fluorescence was also confirmed by GC-MS analyses (26, 31). This removal level is consistent with and only slightly below the previously reported 84% reduction achieved over 10 days. Moreover, *D. hansenii* exhibits higher BaP biotransformation efficiency than other yeasts such as *Candida albicans* (77%), *Rhodotorula mucilaginosa* (70%), and *Saccharomyces cerevisiae* (79%) (31).

The ability of *D. hansenii* to remove BaP reflects a coordinated metabolic adaptation involving the activation of both xenobiotic-transforming and antioxidant enzymes. Key members of these systems, including CYP, CPR, EH, and GST, together with GR, GPx, SOD, and CAT, act in concert with glutathione to maintain intracellular redox balance (11, 20).

The capacity of *D. hansenii* to grow in the presence of BaP without significant loss of viability provides a strong physiological indicator of tolerance to toxic compounds (26, 31, 50–52). Its ability to use BaP as a potential carbon source parallels observations in other fungi such as *Aspergillus* sp. and *Rhodotorula mucilaginosa* (53, 54, 56). Comparable degradation capabilities have been described in yeasts including *Candida guilliermondii*, *Yarrowia lipolytica* (formerly *Candida lipolytica*) (57, 58), *Candida maltosa*, *Candida tropicalis*, *Rhodotorula* spp., *Cryptococcus* spp., and *Debaryomyces* sp. (11). However, the stress tolerance and marine origin of *D. hansenii* make it uniquely suited for studying PAH transformation under glucose limitation. These traits reinforce its potential relevance for environmental and mycoremediation research.

The slower growth rate of *D. hansenii* in the presence of BaP reflects a metabolic adjustment rather than toxicity. Similar to its response under osmotic or saline stress (27, 59), the yeast appears to redirect energy toward biotransformation and redox balance instead of rapid biomass accumulation (11, 56, 60). Fungi from the Ascomycota and Basidiomycota phyla exhibit similar growth patterns, with some species capable of surviving for over 40 days in the presence of anthracene (61), highlighting their capacity to sustain viability through controlled metabolism during prolonged chemical stress.

In *D. hansenii*, BaP metabolism follows the canonical three-phase pathway described for Ascomycete fungi, involving sequential oxidation, hydrolysis, and glutathione conjugation (11, 15). The coordinated induction of ORFs related to detoxification enzymes, including CYP, CPR, EH, and GST, together with their enzymatic activities, coincided with the period of maximum BaP removal, indicating that these processes operate as an integrated detoxification system rather than independent events (26, 31). The basal activity observed in control cells likely reflects their roles in secondary metabolism, gene expression, enzyme activity, and removal kinetics all overlap, which supports the idea of a three-phase detoxification process. Contrary to the notion of isolated events, these phases appear to develop in a coordinated manner (15, 62, 63).

Similar temporal patterns have been reported in other fungi, such as *Aspergillus* species and *R. mucilaginosa*, as well as in previous studies with *D. hansenii*, suggesting that this coupling is a conserved adaptive trait among fungi exposed to PAH. In all cases, the peak expression of CYP genes tends to coincide with the highest rates of BaP removal, suggesting that this phenomenon is a conserved adaptive feature among different fungal species (31, 40, 53, 54, 56). The minimal enzymatic activity observed in YNBG cultures confirms that this response is specifically triggered by BaP rather than constitutive (64).

Among the enzymes induced by BaP, GSTs showed the strongest and most consistent activation at both transcriptional and enzymatic levels. Their activity supports the conjugation of GSH to reactive intermediates, a key step that limits secondary oxidation and channels BaP-derived products toward export or further metabolism (65, 66). In yeast cells, glutathione conjugates are typically sequestered into the vacuole via ABC transporters such as Ycf1p, where they undergo further processing through the γ-glutamyl cycle (67).

Beyond GST, the parallel induction of detoxification- and antioxidant-related genes reflects a coordinated response that mobilizes multiple pathways to preserve redox homeostasis under oxidative stress. This coordination was accompanied by a marked shift in the cellular glutathione balance: GSH levels declined while GSSG accumulated, reducing the GSH/GSSG ratio, a characteristic indicator of oxidative stress (16, 40, 68, 69).

The increased expression of GS indicates an attempt to regenerate the GSH pool, but prolonged exposure likely exhausted this compensatory capacity, revealing a critical threshold in redox control. Although GS is not the rate-limiting enzyme in this pathway (67, 70), its induction highlights the metabolic effort of *D. hansenii* to sustain antioxidant capacity during BaP transformation. The presence of BaP induced a marked oxidative imbalance, evidenced by the rapid accumulation of both cytoplasmic and mitochondrial ROS. This dual localization indicates that oxidative stress arises from multiple cellular compartments, likely linked to oxygen-consuming reactions catalyzed by CYP monooxygenases (11, 17, 18, 71). ROS levels peaked on day 1 and subsequently declined as antioxidant defenses became fully active, yet they remained above basal levels throughout the experiment. Moreover, the addition of exogenous antioxidants and GSH managed to decrease ROS accumulation to baseline levels on day 1 in both compartments (Fig. S2). Concomitant increases in lipid peroxidation, and protein carbonylation confirmed that the oxidative challenge affected both membranes and structural proteins (18, 71–73).

Oxidative patterns similar to those described here have been observed in *S. cerevisiae*, *Dentipellis* sp., *Aspergillus* sp., and *R. mucilaginosa*, where BaP or other PAHs trigger ROS accumulation and the activation of antioxidant defenses (74). Comparable responses have also been reported in extremotolerant yeasts such as *Y. lipolytica* and *Debaryomyces fabryi*, which maintain robust redox protection while metabolizing xenobiotics or growing under challenging conditions (17, 75). Moreover, the presence of analogous mechanisms in organisms such as *Arabidopsis thaliana* and the green alga *Ulva lactuca* (40, 76–78) suggests that the coordination between xenobiotic processing and oxidative stress management is a broadly conserved strategy across eukaryotes.

Following the initial surge in ROS, *D. hansenii* mounted a rapid and well-coordinated enzymatic defense that successfully restored intracellular balance after the second day. The sequential activation of GPx, GR, SOD, and CAT reveals a finely tuned hierarchy of protection, where peroxides and superoxide radicals are detoxified in parallel with ongoing biotransformation reactions. The concurrent decline in both cytoplasmic and mitochondrial ROS highlights an adaptive strategy that efficiently mitigates oxidative by-products while safeguarding cellular redox homeostasis (17, 21, 79).

Noticeably, the transcriptional activation of antioxidant genes preceded the peak of enzymatic activity, particularly in the case of GR, GPx, SOD, and CAT, whose maximum levels were reached 1 day later. A similar time lag between transcript abundance and enzyme activity has been described in other yeasts exposed to oxidative stress, in which mRNA induction is rapid but protein synthesis is delayed due to post-transcriptional control (80–82). Once synthesized, proteins require folding and incorporation of cofactors and coenzymes to achieve their activity (83). Therefore, the delay observed between gene expression and enzymatic activity in *D. hansenii* represents a phase of physiological adjustment that allows the yeast to adapt its antioxidant machinery once the redox imbalance induced by BaP has been detected. 

Redox regulation in *D. hansenii* relies primarily on the glutathione system. The observed shifts in the GSH/GSSG ratio, together with the upregulation of GS and GR, indicate that this pathway plays a dual role, mitigating oxidative stress while sustaining GSH-dependent conjugation reactions during BaP biotransformation. In this context, glutathione metabolism functions as a key interface linking cellular redox regulation with the enzymatic processes of BaP transformation (67, 69, 70).

Beyond its oxidative resilience, *D. hansenii* offers clear advantages over terrestrial degraders such as *Aspergillus*, *Rhizopus*, or *Candida* spp., owing to its halotolerance, nutrient versatility, and capacity to remain metabolically active in saline, low-carbon environments (11, 23, 27). Although its genetic manipulation is still limited, recent development of mutant strains (27) opens the possibility of improving its biodegradation potential through metabolic engineering, microbial consortia, or optimized culture designs. These attributes reinforce *D. hansenii* as a suitable model for BaP transformation and a promising tool for mycoremediation in marine or hypersaline contexts.

While this study highlights BaP oxidation primarily via CYP and EH activity accompanied by ROS accumulation and antioxidant activation, previous reports in *D. hansenii* also described phthalate-like and ring-cleavage metabolites. The concurrent overexpression of short-chain dehydrogenases/reductases, alcohol and aldehyde dehydrogenases, esterases/lipases, and oxidoreductases suggests additional steps that could transform BaP-derived dihydrodiols or conjugates into smaller aliphatic acids that enter central metabolism (26).

**Fig 6:**
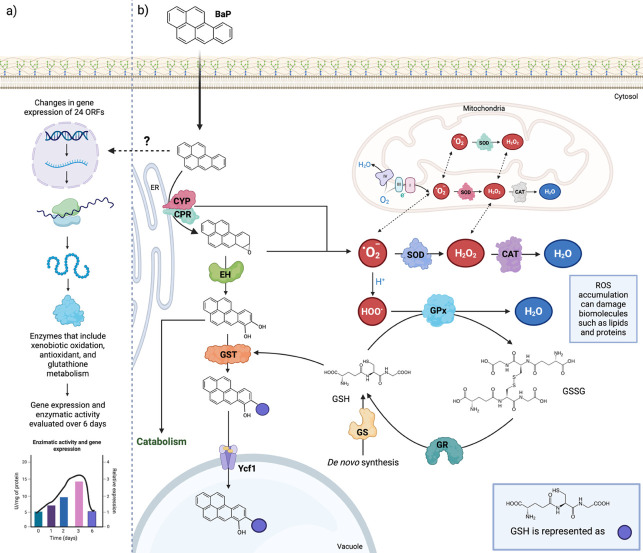
Integrated model of BaP biotransformation, antioxidant responses, and glutathione-dependent detoxification in *D. hansenii*. (**a**) Transcriptomic analysis revealed differential expression of 24 ORFs involved in xenobiotic oxidation, antioxidant defense, and glutathione metabolism. The temporal dynamics of enzymatic activities over 6 days suggested transcriptional induction of these pathways, highlighting a coordinated physiological response to BaP exposure. (**b**) Proposed cellular model for BaP detoxification. Due to its hydrophobic nature, BaP can diffuse across the plasma membrane of *D. hansenii*. Once inside the cell, BaP is oxidized in the endoplasmic reticulum (ER) by cytochrome P450 monooxygenases (CYP) and their associated reductase (CPR), generating reactive intermediates that are further processed by epoxide hydrolase (EH) to form diols that can either be catabolized or conjugated with glutathione (GSH) by glutathione S-transferases (GST). This modification facilitates their transport into the vacuole through the ABC transporter Ycf1. BaP biotransformation initially triggers cytosolic ROS production (largely linked to CYP monooxygenase activity), followed by mitochondrial ROS generation, including superoxide (O_2_•⁻), hydrogen peroxide (H_2_O_2_), and hydroperoxyl radicals (HOO•). These oxidants are removed by superoxide dismutase (SOD), catalase (CAT), and glutathione peroxidase (GPx). Oxidized glutathione (GSSG) is reduced back to GSH by glutathione reductase (GR), while glutathione synthetase (GS) supports *de novo* GSH biosynthesis. The coordination of these processes preserves redox balance and limits oxidative damage to cellular components such as lipids and proteins. The yellow ovals in Ycf1 represent ATP, the purple circle denotes GSH, and the question mark (?) indicates a mechanism that remains unidentified.

These results support an integrative model in which the *xenome* of *D. hansenii*, its inducible genetic and enzymatic repertoire for xenobiotic processing, acts as a dynamically regulated system in response to BaP exposure (11, 15, 84). As illustrated in Fig. 6, the biotransformation cascade involves three phases: aromatic ring oxidation, epoxide hydrolysis, and glutathione conjugation. These pathways are closely linked to antioxidant defenses, particularly the glutathione system.

This coordination indicates that effective BaP transformation in *D. hansenii* relies not only on enzymatic conversion of the compound but also on maintaining cellular redox balance under oxidative stress. The simultaneous modulation of detoxification and antioxidant networks reveals a well-integrated regulatory strategy that promotes survival in chemically challenging environments. The adaptive molecular profile of *D. hansenii*, defined by its broad stress tolerance, highlights its relevance as a eukaryotic model for xenobiotic metabolism. Moreover, its resilience and metabolic versatility underscore its potential for future biotechnological applications, particularly in marine mycoremediation (26, 28, 31).

### Conclusions

*D. hansenii* not only tolerates BaP but also removes it through a response that integrates biotransformation with antioxidant defenses, even under glucose-limiting conditions. The molecular and biochemical evidence confirms the involvement of CYP, CPR, EH, GST, the glutathione system, and major antioxidant enzymes, whose combined activity helps balance the metabolic demands of BaP transformation with the oxidative imbalance generated during the process. Beyond isolated observations, the results indicate that *D. hansenii* couples BaP biotransformation with redox regulation to sustain its viability under chemical stress. Its tolerance to salinity, oxidative conditions, and nutrient limitation further supports its value as a model for studying eukaryotic adaptation and its potential application in mycoremediation strategies in marine environments.

## SUPPLEMENTAL MATERIAL

Figure S1 aem.02314-25-s0001.pdf RT-qPCR data used to construct the heat maps shown in the article.

Figure S2 aem.02314-25-s0002.pdf Antioxidant supplementation reverses BaP-induced cytoplasmic and mitochondrial ROS accumulation in *D. hansenii* at 24 h.

Table S1 aem.02314-25-s0003.docx Oligonucleotides used in RT-qPCRs.

Table S2 aem.02314-25-s0004.docx Function of 24 upregulated genes in *D. hansenii* associated with BaP detoxification, glutathione metabolism, and antioxidant defense.

ASM does not own the copyrights to Supplemental Material that may be linked to, or accessed through, an article. The authors have granted ASM a non-exclusive, world-wide license to publish the Supplemental Material files. Please contact the corresponding author directly for reuse.
