# Supplementary material for: Mechanisms of the marine yeast Debaryomyces hansenii for protection against reactive oxygen species produced during benzo(a)pyrene biotransformation
Source: Appl Environ Microbiol. 2026 Jan 7;92(2):e02314-25. doi: 10.1128/aem.02314-25 (PMC12915302; doi:10.1128/aem.02314-25)
Supplement: Figure S1 — RT-qPCR data used to construct the heat maps shown in the article. [file aem.02314-25-s0001.pdf]

Supplementary Figure 1. RT-qPCR data used to construct the heat maps shown in the article.

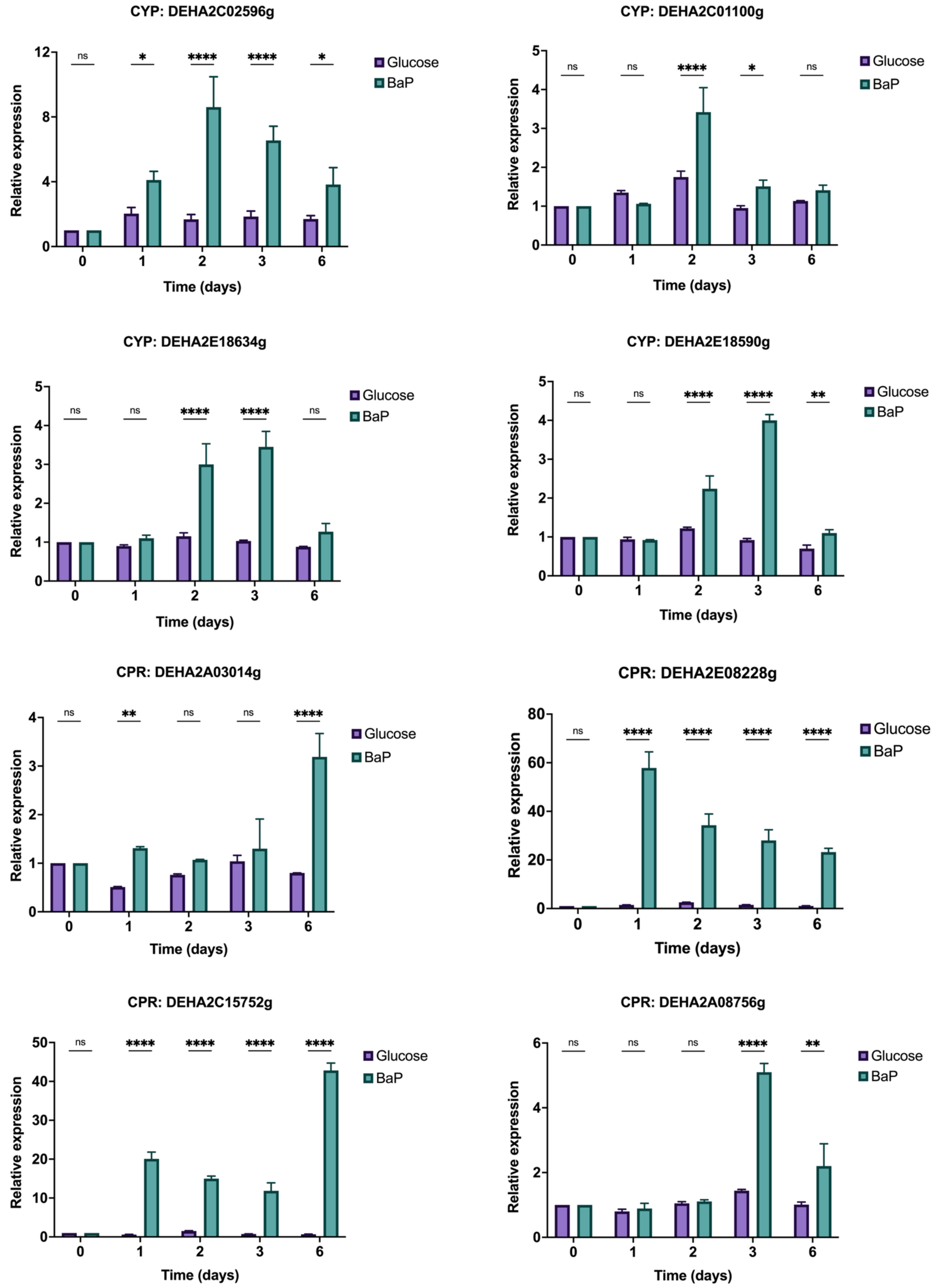

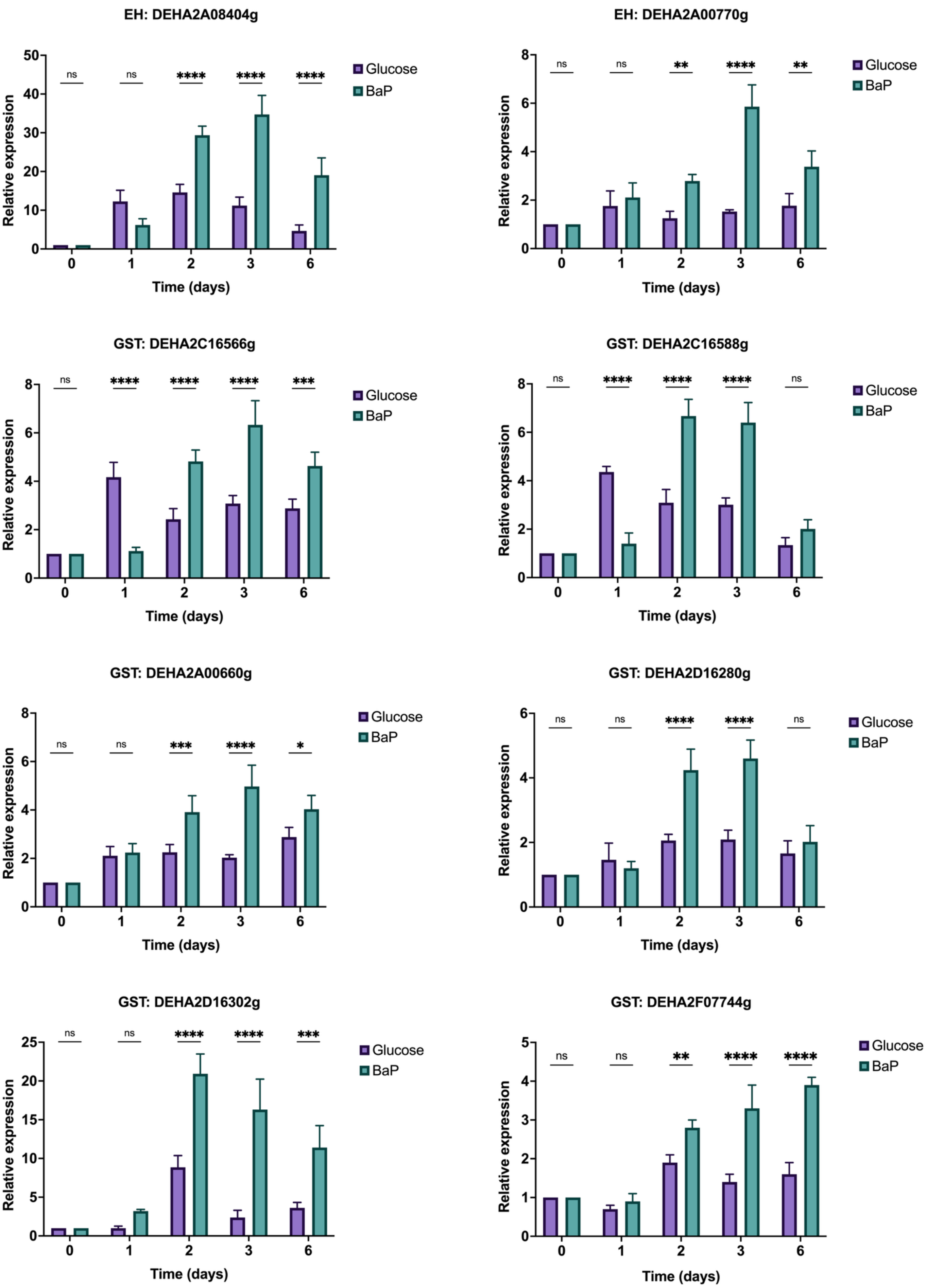
EH: DEHA2A08404g
Relative expression
Time (days)
Glucose
BaP
ns ns **** **** ****
EH: DEHA2A00770g
Relative expression
Time (days)
Glucose
BaP
ns ns ** **** **
GST: DEHA2C16566g
Relative expression
Time (days)
Glucose
BaP
ns **** **** **** ***
GST: DEHA2C16588g
Relative expression
Time (days)
Glucose
BaP
ns **** **** **** ns
GST: DEHA2A00660g
Relative expression
Time (days)
Glucose
BaP
ns ns *** **** *
GST: DEHA2D16280g
Relative expression
Time (days)
Glucose
BaP
ns ns **** **** ns
GST: DEHA2D16302g
Relative expression
Time (days)
Glucose
BaP
ns ns **** **** ***
GST: DEHA2F07744g
Relative expression
Time (days)
Glucose
BaP
ns ns ** **** ****

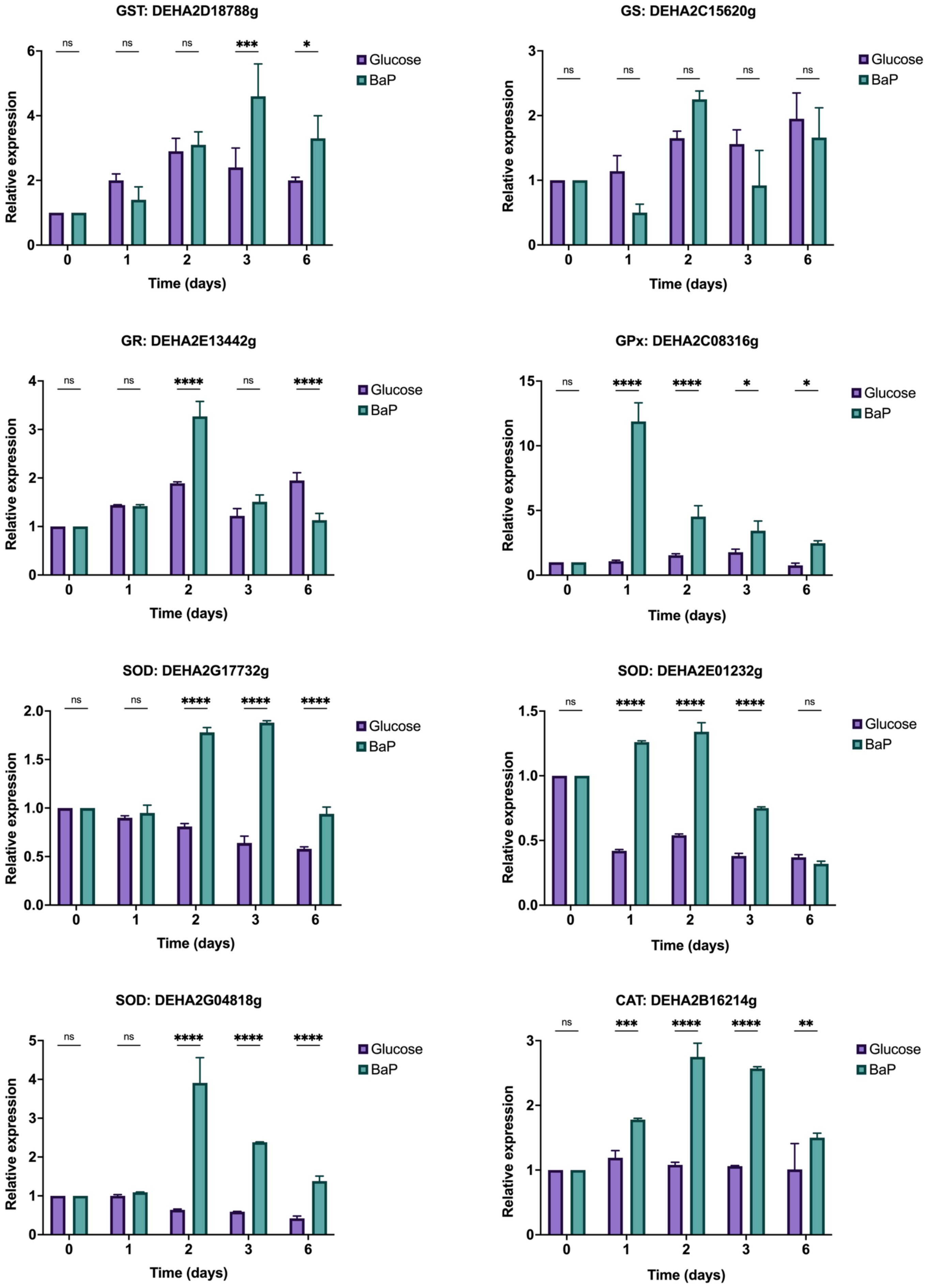

Data are shown as mean ± SD from four independent biological replicates, each measured in technical duplicate. Statistical significance was determined by two-way ANOVA with a full factorial model and Šidák's post hoc correction for multiple comparisons ($\alpha = 0.05$). Adjusted P values were reported as follows: ns ($P \geq 0.1234$), * ($P < 0.0332$), ** ($P < 0.0021$), *** ($P < 0.0002$), and **** ($P < 0.0001$).
