# Supplementary material for: Mechanisms of the marine yeast Debaryomyces hansenii for protection against reactive oxygen species produced during benzo(a)pyrene biotransformation
Source: Appl Environ Microbiol. 2026 Jan 7;92(2):e02314-25. doi: 10.1128/aem.02314-25 (PMC12915302; doi:10.1128/aem.02314-25)
Supplement: Figure S2 — Antioxidant supplementation reverses BaP-induced cytoplasmic and mitochondrial ROS accumulation in D. hansenii at 24 h. [file aem.02314-25-s0002.pdf]

Supplementary Figure 2. Antioxidant supplementation reverses BaP-induced cytoplasmic and mitochondrial ROS accumulation in *D. hansenii* at 24 h.

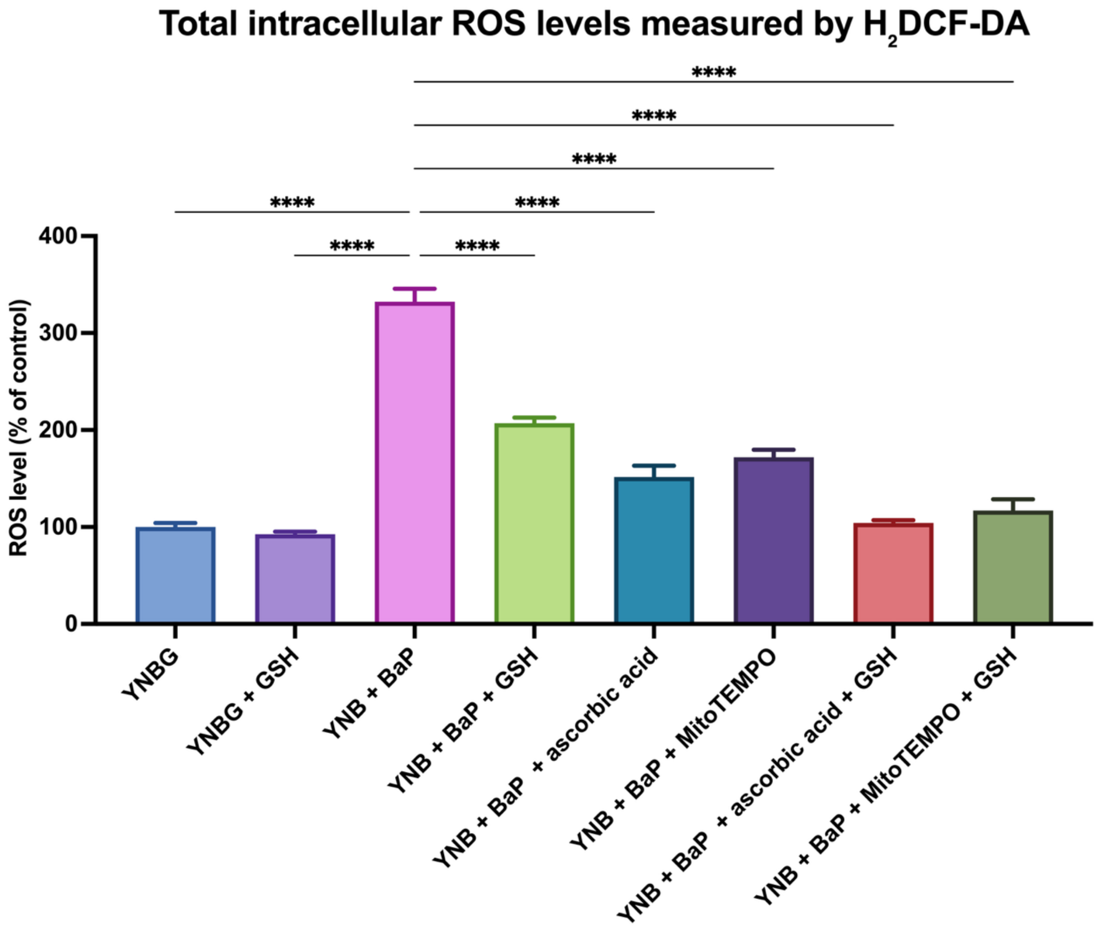

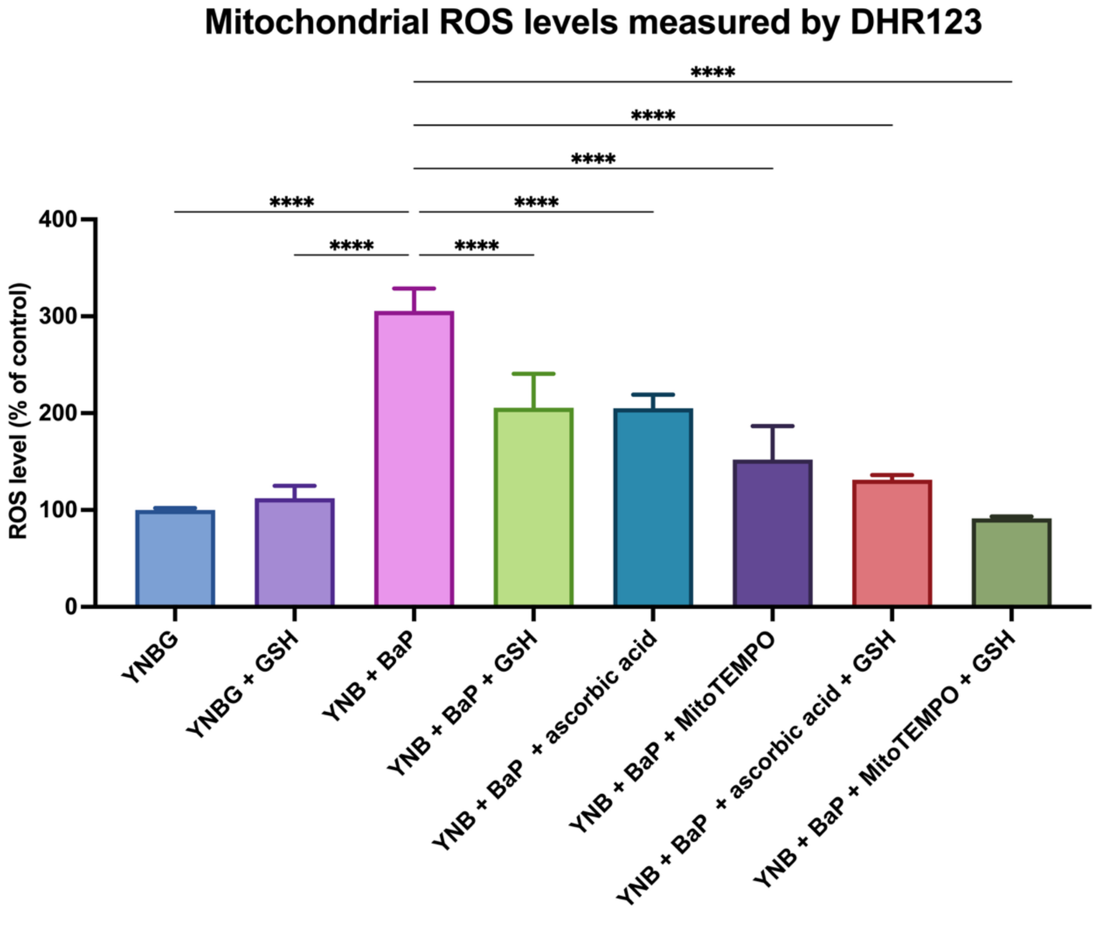

Quantification of cytoplasmic and mitochondrial ROS in *D. hansenii* cultures exposed to BaP and antioxidant combinations at 24 h. To clarify the cellular origin of BaP-induced oxidative stress, total (cytoplasmic) and mitochondrial ROS were re-measured at the 24-h time point, which corresponded to the maximal accumulation detected in the time-course analysis (see Fig. 4). Cultures were grown in YNB medium under the following conditions: control (YNBG), YNBG + GSH (2.5 mM), YNB + BaP (100 ppm), YNB + BaP + GSH (2.5 mM), YNB + BaP + ascorbate (1 mM), YNB + BaP + MitoTEMPO (1 μM), and combinations of BaP with GSH plus either ascorbate or MitoTEMPO. Total ROS were quantified using H_2DCF-DA, whereas mitochondrial ROS were quantified using DHR123, whose accumulation depends on the mitochondrial membrane potential.
