# Supplementary material for: Mechanisms of the marine yeast Debaryomyces hansenii for protection against reactive oxygen species produced during benzo(a)pyrene biotransformation
Source: Appl Environ Microbiol. 2026 Jan 7;92(2):e02314-25. doi: 10.1128/aem.02314-25 (PMC12915302; doi:10.1128/aem.02314-25)
Supplement: Table S1 — Oligonucleotides used in RT-qPCRs. [file aem.02314-25-s0003.docx]

**Supplementary Table 1. Oligonucleotides used in RT-qPCRs**

| **ORF ID** | **Primer** | **Sequence** |
| --- | --- | --- |
| **DEHA2D05412g**  ***ACT1*** | Forward | CCCAGAAGAACACCCAGTTT |
|  | Reverse | CGGCTTGGATAGAAACGTAGAA |
| **DEHA2C02596g** | Forward | GCGTTGGGTACAAGACATGC |
|  | Reverse | CTGTTCACGTGCAAACTGGG |
| **DEHA2C01100g** | Forward | CGATGCAGCAACGGAGTTTT |
|  | Reverse | TACAAATCCTGCGCCAAAGC |
| **DEHA2E18634g** | Forward | TCGGAGAGGGAGAAGATTCCA |
|  | Reverse | TTGGCGATTTTCCATCGGGA |
| **DEHA2E18590g** | Forward | TTGGTGCGAAACCAATGCAG |
|  | Reverse | CATCCCGCGTAACTACAGCA |
| **DEHA2A03014g** | Forward | AAATCGACGGAAAGGACGAG |
|  | Reverse | AGCATGGCAAATCTCTTGGA |
| **DEHA2E08228g** | Forward | ACAAGGCTCCTGAAAACTGG |
|  | Reverse | GGACCACAGACGAAAACCTT |
| **DEHA2C15752g** | Forward | GGGTGGTCATGGTGTTTACC |
|  | Reverse | TCCCATTGCTTGCCATACTT |
| **DEHA2A08756g** | Forward | GGTGCACTTGGTATGATGGT |
|  | Reverse | CTGGAACAAGTCTCCAGCAA |
| **DEHA2A00770g** | Forward | CTGCTGGTCTCCGACATGGA |
|  | Reverse | AAGTCTACGTTCCAGCGCCT |
| **DEHA2A08404g** | Forward | AGTCAGAAGCCTCGACAGGG |
|  | Reverse | TGTTTCTGAGAACGCAGCCA |
| **DEHA2C16566g** | Forward | AGACCGGTTTCTCTACTAAGCAAG |
|  | Reverse | ATTCGATTTTGTTAGGACCTTCGT |
| **DEHA2C16588g** | Forward | TTTGGCCAAGAACAAAGAAAGTT |
|  | Reverse | TGGACATAAACGGCATCGAA |
| **DEHA2A00660g** | Forward | TCCACGCAATTCTTCCAGGT |
|  | Reverse | ACCTTGAAAGATCGATGCTCCTGT |
| **DEHA2D16280g** | Forward | AGGCCCTCGACTTTTTGGAA |
|  | Reverse | CCGCCAGTCATAGCCTTTGT |
| **DEHA2D16302g** | Forward | AGAAAGGGAGGGGACAGGTT |
|  | Reverse | CACCATGCAGCAAGATGTGG |
| **DEHA2D18788g** | Forward | TTGTTAAGTTCGCGCCTGAG |
|  | Reverse | ACTTGGCCCACGAATAGACG |
| **DEHA2F07744g** | Forward | GCGGCCGCTTATAGTGCTG |
|  | Reverse | GCCACAGTAGCTCTATCACCAACT |
| **DEHA2E13442g** | Forward | ACCTTAACCGGAGACCAAGA |
|  | Reverse | TAGCATATCCTCCTGTGGCA |
| **DEHA2C08316g** | Forward | GAGAGGGGATTTGTTGTGCT |
|  | Reverse | TCCGTTGCTCGTTCTTCAAA |
| **DEHA2C15620g** | Forward | ACTCACAGTGCTACACATGC |
|  | Reverse | AGTCATCGTCAAAAACGGCT |
| **DEHA2G17732g** | Forward | CGGTGTCGTTAACTTCGAACA |
|  | Reverse | TGAACGGGTTAAAGTGAGGT |
| **DEHA2D01232g** | Forward | GGCAAGGAAGCTCATAGCGA |
|  | Reverse | CTGGATCATCAGGCCCGTC |
| **DEHA2G04818g** | Forward | CGTCGGACTCTGAATTGGCA |
|  | Reverse | TGGTCCCGTAACAGTGTCTTG |
| **DEHA2B16214g** | Forward | CATAYGGTGCCCAAACGGC |
|  | Reverse | ACCACCACCTTTTGCGTGAA |
