# Supplementary material for: Mechanisms of the marine yeast Debaryomyces hansenii for protection against reactive oxygen species produced during benzo(a)pyrene biotransformation
Source: Appl Environ Microbiol. 2026 Jan 7;92(2):e02314-25. doi: 10.1128/aem.02314-25 (PMC12915302; doi:10.1128/aem.02314-25)
Supplement: Table S2 — Function of 24 upregulated genes in D. hansenii associated with BaP detoxification, glutathione metabolism, and antioxidant defense. [file aem.02314-25-s0004.docx]

**Supplementary Table 2. Function of 24 upregulated genes in *D. hansenii* associated with BaP detoxification, glutathione metabolism, and antioxidant defense.**

| **ORF ID** | **Function** |
| --- | --- |
| **DEHA2C02596g** | Encodes a cytochrome P450 monooxygenase (CYP4/CYP19/CYP26 family) involved in oxidative metabolism of hydrophobic compounds. Contains heme- and iron-binding domains (PF00067) and catalyzes reactions incorporating oxygen into substrates, likely contributing to xenobiotic detoxification. |
| **DEHA2C01100g** | Encodes a cytochrome P450 monooxygenase (CYP4/CYP19/CYP26 family) with heme- and iron-binding domains (PF00067). Functions in oxidative metabolism and detoxification by incorporating oxygen into hydrophobic substrates. |
| **DEHA2E18634g** | Encodes a cytochrome P450 monooxygenase (CYP4/CYP19/CYP26 family) involved in oxidation of hydrophobic compounds. Features heme- and iron-binding domains (PF00067) and catalyzes oxygen incorporation into diverse substrates, supporting detoxification pathways. |
| **DEHA2E18590g** | Encodes a cytochrome P450 monooxygenase (CYP4/CYP19/CYP26 family) with roles in oxidative transformation of hydrophobic substrates. Contains conserved heme- and iron-binding domains (PF00067), supporting functions in xenobiotic metabolism and detoxification. |
| **DEHA2A03014g** | Encodes a cytochrome b5 reductase (KOG0534; EC 1.6.2.2) with conserved FAD- and NAD-binding domains (PF00970, PF00175). Participates in electron transfer reactions, likely contributing to lipid metabolism and redox homeostasis. |
| **DEHA2E08228g** | Encodes a cytochrome b5 reductase (KOG0534; EC 1.6.2.2) containing FAD- and NAD-binding domains (PF00970, PF00175). Functions as an oxidoreductase in electron transfer reactions, possibly linked to membrane-associated redox processes. |
| **DEHA2C15752g** | Encodes a membrane-bound Δ14-sterol reductase (KOG1435; EC 1.3.1.70) of the ERG4/ERG24 family (PF01222), involved in ergosterol biosynthesis. Essential for maintaining membrane structure and sterol composition. |
| **DEHA2A08756g** | Encodes a membrane-associated Δ24(24¹)-sterol reductase (KOG1435; EC 1.3.1.71) of the ERG4/ERG24 family (PF01222), involved in the final steps of ergosterol biosynthesis. Contributes to membrane integrity and sterol homeostasis. |
| **DEHA2A00770g** | Encodes a soluble epoxide hydrolase (KOG4178; EC 3.3.2.10) with an alpha/beta hydrolase fold (PF00561), involved in hydrolyzing epoxides to less reactive diols. Contributes to detoxification of lipid-derived or xenobiotic epoxides. |
| **DEHA2A08404g** | Encodes a soluble epoxide hydrolase (KOG4178) with alpha/beta hydrolase fold (PF00561), likely involved in the detoxification of epoxide-containing compounds through hydrolysis. Supports cellular protection against reactive intermediates. |
| **DEHA2C16566g** | Encodes a predicted glutathione S-transferase (KOG2903) with conserved GST-related domains (IPR010987, IPR012336), likely involved in glutathione-dependent detoxification and protection against oxidative damage. |
| **DEHA2C16588g** | Encodes a predicted glutathione S-transferase (KOG2903) with conserved domains (IPR010987, IPR012336) likely involved in detoxification through glutathione conjugation. Contributes to cellular defense against oxidative and chemical stress. |
| **DEHA2A00660g** | Encodes a glutathione S-transferase (KOG0868) involved in conjugating glutathione to a variety of toxic compounds. Contains a conserved N-terminal GST domain (PF02798), contributing to detoxification and stress resilience. |
| **DEHA2D16280g** | Encodes a glutathione S-transferase (KOG0867) that facilitates the conjugation of glutathione to electrophilic substrates. Includes conserved GST N- and C-terminal domains (PF02798, PF00043), supporting roles in detoxification and oxidative stress response. |
| **DEHA2D16302g** | Encodes a glutathione S-transferase (KOG0867) involved in cellular detoxification via glutathione conjugation. Contains conserved GST N- and C-terminal domains (PF02798, PF00043), essential for xenobiotic defense and oxidative stress adaptation. |
| **DEHA2D18788g** | Encodes a glutathione S-transferase (KOG0867) that catalyzes the conjugation of glutathione to toxic compounds. Features conserved N- and C-terminal GST domains (PF02798, PF00043), supporting roles in detoxification and cellular stress response. |
| **DEHA2F07744g** | Encodes a glutathione S-transferase (KOG0867) involved in detoxification through conjugation of glutathione to electrophilic compounds. Contains conserved N- and C-terminal GST domains (PF02798, PF00043) critical for xenobiotic metabolism and stress defense. |
| **DEHA2E13442g** | Encodes a glutathione-disulfide reductase (KOG0405; PF00070) that uses NADPH to regenerate reduced glutathione (GSH). Involved in redox homeostasis and antioxidant defense, with conserved FAD and NADP-binding domains. |
| **DEHA2C08316g** | Encodes a glutathione peroxidase (KOG1651; PF00255) that reduces H_2_O_2_ and lipid peroxides using glutathione. Plays a key role in oxidative stress response and cellular redox homeostasis. |
| **DEHA2C15620g** | Encodes a glutathione synthase (KOG0021) involved in the final step of glutathione biosynthesis. Contains ATP-binding and catalytic domains (PF03199, PF03917), essential for maintaining redox homeostasis and antioxidant defense. |
| **DEHA2G17732g** | Encodes a Cu/Zn superoxide dismutase (SOD1; KOG0441) that catalyzes the detoxification of superoxide radicals. Contains the conserved PF00080 domain and requires copper and zinc ions as cofactors to maintain redox balance under oxidative stress. |
| **DEHA2D01232g** | Contains conserved Mn/Fe-binding domains (PF02777, PF00081), essential for ROS detoxification during oxidative stress. |
| **DEHA2G04818g** | Encodes a manganese-dependent superoxide dismutase (SOD; KOG0876) that catalyzes the conversion of superoxide radicals to H_2_O_2_ and oxygen. Contains conserved Mn/Fe-binding domains (PF02777, PF00081), essential for ROS detoxification during oxidative stress. |
| **DEHA2B16214g** | Encodes a catalase (PF00199) that decomposes H_2_O_2_ into water and oxygen. Plays a key role in oxidative stress response by preventing reactive oxygen species (ROS) accumulation. |

List of 24 open reading frames (ORFs) identified as significantly up-regulated in *D. hansenii* on day 3 of exposure to BaP, based on RNA-Seq data ((NCBI Gene Expression Omnibus database under accession No. GSE299919 (Padilla-Garfias et al. 2025)). Gene functions were assigned using MycoCosm gene ontology annotations (Grigoriev et al. 2014). These ORFs include detoxification enzymes, antioxidant proteins, and key regulators of glutathione homeostasis. Full RT-qPCR data are shown in Supplementary Figure 1; primer sequences are listed in Supplementary Table 1.
